# Piperine, quercetin, and curcumin identified as promising natural products for topical treatment of cutaneous leishmaniasis

**DOI:** 10.1007/s00436-024-08199-w

**Published:** 2024-04-18

**Authors:** Camila M. Clemente, Javier Murillo, Ariel G. Garro, Natalia Arbeláez, Tatiana Pineda, Sara M. Robledo, Soledad Ravetti

**Affiliations:** 1 Departamento de Química Biológica, Facultad de Ciencias Exactas y Naturales, Universidad de Buenos Aires (FCEyN-UBA) E Instituto de Química Biológica de La Facultad de Ciencias Exactas y Naturales (IQUIBICEN) CONICET, Ciudad de Buenos Aires, C1428EHA Argentina; 2 Grupo Estudios Preclínicos Para El Desarrollo de Productos, Corporación de Innovación CIDEPRO, Medellín, Colombia; 3 PECET-Facultad de Medicina, Universidad de Antioquia, Calle 70 # 52-21, Medellín, Colombia; 4 Ministerio de Ciencia y Tecnología de La Provincia de Córdoba (MinCyT-CBA), Álvarez de Arenales 230, CP 5004 Córdoba, Argentina; 5 Instituto Académico Pedagógico de Ciencias Humanas, Universidad Nacional de Villa María, Campus Universitario, Av. Arturo Jauretche 1555, CP 5900 Villa María, Argentina; 6 Centro de Investigaciones y Transferencia de Villa María (CIT VM), CP 5900 Villa María, Argentina

**Keywords:** *Leishmania*, *L. braziliensis*, Topical treatment, Antileishmanial drug, Natural compound

## Abstract

*Leishmania braziliensis* (*L. braziliensis*) causes cutaneous leishmaniasis (CL) in the New World. The costs and the side effects of current treatments render imperative the development of new therapies that are affordable and easy to administer. Topical treatment would be the ideal option for the treatment of CL. This underscores the urgent need for affordable and effective treatments, with natural compounds being explored as potential solutions. The alkaloid piperine (PIP), the polyphenol curcumin (CUR), and the flavonoid quercetin (QUE), known for their diverse biological properties, are promising candidates to address these parasitic diseases. Initially, the in vitro cytotoxicity activity of the compounds was evaluated using U-937 cells, followed by the assessment of the leishmanicidal activity of these compounds against amastigotes of *L. braziliensis*. Subsequently, a golden hamster model with stationary-phase *L. braziliensis* promastigote infections was employed. Once the ulcer appeared, hamsters were treated with QUE, PIP, or CUR formulations and compared to the control group treated with meglumine antimoniate administered intralesionally. We observed that the three organic compounds showed high in vitro leishmanicidal activity with effective concentrations of less than 50 mM, with PIP having the highest activity at a concentration of 8 mM. None of the compounds showed cytotoxic activity for U937 macrophages with values between 500 and 700 mM. In vivo, topical treatment with QUE daily for 15 days produced cured in 100% of hamsters while the effectiveness of CUR and PIP was 83% and 67%, respectively. No failures were observed with QUE. Collectively, our data suggest that topical formulations mainly for QUE but also for CUR and PIP could be a promising topical treatment for CL. Not only the ease of obtaining or synthesizing the organic compounds evaluated in this work but also their commercial availability eliminates one of the most important barriers or bottlenecks in drug development, thus facilitating the roadmap for the development of a topical drug for the management of CL caused by *L. braziliensis*.

## Introduction

Cutaneous Leishmaniasis (CL) is a neglected tropical disease that is a major public health issue in several countries worldwide. It predominantly affects people of low socioeconomic status. It is the second most deadly vector-transmitted disease with over 1 billion people living in endemic areas and at risk of infection according to the World Health Organization (WHO 2023). More than 20 *Leishmania* species infect humans; they are transmitted during the blood meal of female phlebotomine sandflies. Infection can cause a wide spectrum of clinical manifestations including plaques, nodules, ulcers, and crusty lesions (WHO 2023). CL has an estimated global incidence of 700,000 to one million cases each year. Although not fatal, CL has profound socio-economic impacts due to the stigmatization of infected and cured individuals, as these latter may bear disfiguring scars (WHO 2023). Currently, recommendations for CL treatment depend on the country where the patient is infected, the clinical form of the disease, and the infecting *Leishmania* spp. Systemic therapies such as intramuscular or intravenous administration of pentavalent antimonials, oral administration of miltefosine, or intramuscular administration of pentamidine isethionate, as well as local therapies such as intralesional administration of pentavalent antimonials and topical application of drugs such as paromomycin are available for the treatment of CL (WHO 2023). Indeed, management guidelines recently published by the Pan American Health Organization (PAHO) begin to recommend the use of local therapies such as thermotherapy and intralesional application of pentavalent antimony (OPS 2013), which leaves the door open to include new drugs for topical application that are currently under development and clinical evaluation. Local therapies are recommended for patients with a low number of lesions. To this end, there has been a growing interest in developing safe, affordable, non-parenteral efficacious topical treatments. The study cited by Azim et al. (2021) underscores the utilization of ointments and creams containing various compounds such as paromomycin, imiquimod, amphotericin B, and combinations of drugs in the treatment of cutaneous leishmaniasis lesions. Clinical trials have yielded promising outcomes, demonstrating notable rates of recovery among patients afflicted with cutaneous leishmaniasis (Azim et al. 2021).

Departing from conventional treatments, natural compounds have emerged as pivotal alternatives in therapeutic strategies. Their inherent properties, derived from botanical sources, offer a promising avenue for exploring novel treatment modalities with potentially fewer side effects and enhanced efficacy. Aligned with these principles, researchers and healthcare professionals are actively engaged in the quest for novel therapeutic compounds and the development of safe, affordable, and effective innovative topical treatments for CL. Safety remains paramount in these endeavors, ensuring that treatments are easily accessible, straightforward to administer, and adaptable for use in endemic regions without incurring exorbitant costs or necessitating frequent clinical visits. This multidisciplinary and patient-centric approach is imperative for effectively addressing the burden of CL and enhancing the quality of life for affected individuals.

**Fig. 1:**
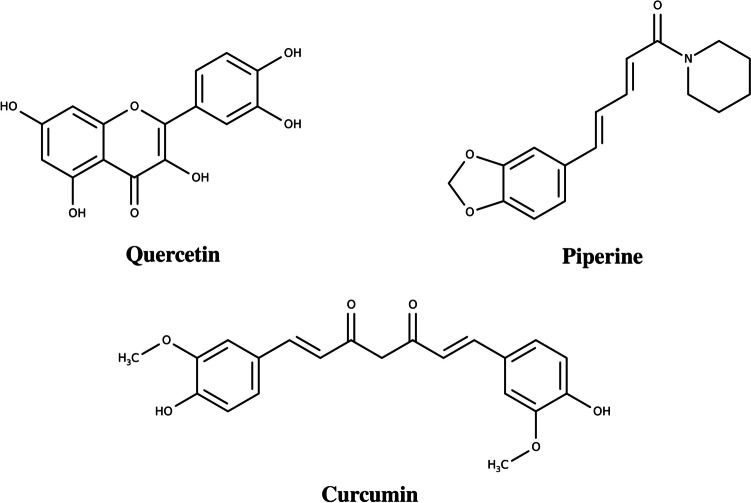
Chemical structure of quercetin (QUE), piperine (PIP), and curcumin (CUR)

Quercetin (QUE), piperine (PIP), and curcumin (CUR) are three natural compounds that have garnered significant attention in scientific research due to their potential health benefits (Boots et al. 2008; Aggarwal and Harikumar 2009; Moorthi and Kathiresan 2013; Khan et al. 2019; Haq et al. 2021; Zou et al. 2021). In terms of their chemical similarities (Fig. 1), QUE and CUR are polyphenols found in a variety of plant-based foods, while PIP is an alkaloid present in black pepper. Despite these structural differences, all three compounds have demonstrated antioxidant, anti-inflammatory, antimicrobial, and anticancer properties in scientific studies (Hussain et al. 2017; Haq et al. 2021; Azeem et al. 2023).

When considering the demonstrated biological differences among them, QUE has been shown to reduce the risk of heart diseases (Papakyriakopoulou et al. 2022) and diabetes (Roshanravan et al. 2023). CUR has displayed beneficial effects in pain relief (Uddin et al. 2021) and the progression of anti-viral processes (Thimmulappa et al. 2021). Furthermore, PIP has been reported to possess immunomodulatory properties (Sunila and Kuttan 2004) and has been scientifically validated for its capacity to enhance the absorption of other drugs and nutrients (Saini et al. 2023), including CUR (Gervazoni et al. 2020).

QUE has demonstrated a decrease of the *Leishmania* parasitic load through different mechanisms. These mechanisms include inhibiting the oxidation of proteins and lipids on the red cell membranes of infected animals (Fonseca-Silva et al. 2011), direct interference with mitochondrial physiology (Fonseca-Silva et al. 2011), interaction with DNA topoisomerases (Sarkar et al. 2002), or with different active sites of essential proteins for the survival of *Leishmania* spp. (Gervazoni et al. 2020). In turn, CUR and its derivatives have demonstrated significant leishmanicidal activity, both in vitro and in vivo, against amastigotes and promastigotes of different *Leishmania* species (Albalawi et al. 2021a). To date, research has confirmed CUR ability to inhibit the action of nitric oxide (NO), thereby overcoming the inhibitory effect of NO on *Leishmania* spp. (Das et al. 2008). Furthermore, studies have indicated that a CUR analog plays a role in regulating the balance between autophagy and apoptosis (Basmaciyan et al. 2018). Additionally, combining CUR with other first-line drugs for leishmaniasis, such as miltefosine, has been shown to enhance the effectiveness of these treatments (Rasmussen et al. 2000; Chauhan et al. 2018). Lastly, PIP has also been documented for its antiprotozoal potential against various parasites, including *Leishmania* spp. (Kapil 1993; Ferreira et al. 2011) and *Trypanosoma cruzi* (Rani et al. 2020). In addition, PIP has been used to enhance the bioavailability of several drugs, such as amphotericin B in *L. donovani* (Ray et al. 2021). The absorption, distribution, metabolism, excretion, and toxicity of a therapeutic agent could be enhanced by combining it with bioenhancers. Studies have demonstrated that PIP enhances the antimalarial activity of CUR in *Plasmodium berghei* (Khairani et al. 2022).

In the present study, we evaluate the leishmanicidal activity of CUR, QUE, and PIP in the in vitro model of intracellular amastigotes obtained after the infection of the U937 macrophages with *L. braziliensis*; in addition, we investigated the outcome of topical formulations of these natural compounds, using the experimental hamster model of *L. braziliensis*-induced CL.

## Materials and methods

### Compounds

QUE, PIP, and CUR were purchased from Sigma-Aldrich (Buenos Aires, Argentina). For the in vitro studies, compounds were dissolved in dimethyl sulfoxide (DMSO, Sigma), ensuring that the final concentration of DMSO remained below 0.5%, a level considered non-toxic for both cells and parasites. Six serial dilutions (1:4) of derivatives tested (700, 175, 43.8, 10.9, 2.7, and 0.6 × µg/mL) were prepared in RPMI 1640 (Sigma-Aldrich) media supplemented with 10% heat-inactivated fetal bovine serum (FBS, Invitrogen). Internal controls for cytotoxicity and leshmanicidal activity included doxorubicin (Santa Cruz Biotechnology), a drug used to treat several cancers, and amphotericin B (Merck), a drug used to treat leishmaniasis. Both controls were tested at concentrations of 1, 0.5, 0.25, 0.125, 0.06, and 0.03 µM. The stock solutions were kept at 4 °C and fresh serial dilutions were made for every assay. 2,2-diphenyl-1-picrylhydrazyl (DPPH) and 3-(4,5-dimethylthiazol-2-yl)-2,5-diphenyl tetrazolium bromide) (MTT) were obtained from Sigma-Aldrich.

### QUE, CUR, and PIP formulations for in vivo studies

An emulsion formulation containing 1, 1.5, or 2.5 mg of QUE, CUR, and PIP, respectively, in 600 L of polyoxyl castor oil was used for in vivo treatment.

### Cells and cytotoxicity

U-937 (CRL-1593.2™) macrophages (ATCC, Manassas, VA, USA) were cultured in RPMI-1640 medium supplemented with 10% of FBS and incubated at 37 °C in 5% CO_2_, 95% humidity atmosphere. U-937 cells in the exponential growth phase were recovered, and washed by centrifugation at 1800 rpm for 10 min at room temperature; the pellet was then resuspended in supplemented RPMI 1640 medium and counted in a Neubauer chamber. The number was adjusted to 1 × 10^6^ cells per mL and 100 µL was dispensed into each well of a 96-well plate. Then, another 100 µL of each serial dilution of QUE, PIP, CUR, doxorubicin, and amphotericin B was added to each well by triplicate and plates were incubated at 37 °C 5% CO2, 95% humidity atmosphere for 48 h.

The MTT solution is then added to the treated cells, where the yellow MTT is reduced to purple formazan by a variety of mitochondrial and cytosolic enzymes that are operational in viable cells. After 3 h of incubation with MTT, the formazan precipitates. Consequently, the growing medium was removed, the formazan was dissolved in DMSO, and the absorbance of the solution was measured at 570 nm using a microplate reader spectrophotometer (Varioskan Flash, Thermo Fisher Scientific).

Cells exposed to the standard cytotoxic drug doxorubicin were used as an internal control for toxicity (positive control) while cells incubated in the absence of any compound or drug were used as a control for viability (negative control). A solution of 0.5% DMSO was used as a blank solution. Non-specific absorbance was corrected by subtracting absorbance (OD) of the blank. Determinations were done by triplicate in at least two independent experiments.

The OD of viable cells that remained after treatment was compared to the OD of control cells that were not exposed to the drug. The LC_50_ (median lethal concentration) values and their statistical errors were calculated based on triplicates of the measurement in the independent assays. The LC_50_ values were determined by non-linear regression using Prism GraphPad 10.

### Parasites and in vitro leishmanicidal activity of CUR, PIP, and QUE in infected U-937 macrophages

Infective *L. braziliensis* (MHOM/CO/88/UA301-eGFP) (Pulido et al. 2012) were cultured in Novy-MacNeal-Nicolle (NNN) biphasic medium at 26 °C for 3 passages maximum. U-937 cells, prepared as described in the previous section, were adjusted to approximately 3 × 10^5^ cells per mL of supplemented RPMI 1640 medium containing 0.1 ng/ml of PMA to induce cell adherence. One mL was dispensed into each well of a 24-well plate and platters were incubated at 37 °C in % CO_2_, 95% humidity atmosphere for 72 h. Then, adherent cells were infected with fluorescent promastigotes of *L. braziliensis* in a 15:1 parasite to cell ratio. Plates were incubated again at 34 °C and % CO_2_, 95% humidity atmosphere. After 24 h, non-phagocytosed parasites were washed by pipetting phosphate buffered solution (PBS) in the plate at room temperature and 500 µL of fresh complete RPMI 1640 medium was added. Then, 500 µL of each serial dilution of QUE, PIP, CUR, and amphotericin B was added to each well and plates were incubated at 34 °C with % CO_2_, 95% humidity atmosphere. The EC_50_, the median effective concentration to induce maximal killing of the intracellular amastigotes in infected cells, was measured by Flow cytometry at 488 nm of excitation and 525 nm of emission. After 48 h of incubation, cells were harvested with cold dissociation buffer (1x PBS with 2% EDTA) and the harvested cells were washed with buffer (1 × PBS with 2% FBS), and results were analyzed by FlowJo 10. The EC_50_ was determined with a non-lineal regression using Prism GraphPad 10.

### In vivo therapeutic response of QUE, PIP, and CUR in infected golden hamsters

Golden hamsters (*Mesocricetus auratus*) were purchased from Charles River Laboratories (Wilmington MA, USA) and bred under specific pathogen-free conditions at the animal facility unit of the Universidad de Antioquia in Medellín, Colombia. Six- to eight-week-old, male, and female hamsters were used for the experiments. Animal experimentation protocols including handling of the animals, sample collection, and euthanasia were done in compliance of the Center for Diseases Control and Prevention guidelines for Safe Work Practices in Human and Animal Medical Diagnostic Laboratories and ARRIVE guidelines (Percie du Sert et al. 2020b; Khairani et al. 2022) and approved by the Institutional Ethical Committee for animal experimentation (Act No. 152 of June 01, 2023) (Percie du Sert et al. 2020a; Khairani et al. 2022).

Hamsters were anesthetized with ketamine (80 mg/kg, i.p.) and xylazine (15 mg/Kg) before infection and then, ∼1 × 10^8^ promastigotes in stationary phase (6 days-old growth) were inoculated into the dorsum dermis (Robledo et al. 2012). When hamsters developed ulcers larger than 12.5 mm^2^, hamsters were randomly distributed into 4 groups (*n* = 6, each): (1) group treated with QUE (1 mg/day), (2) group treated with PIP (2.5 mg/day), (3) group treated with CUR (1.5 mg/day), (4) group treated with meglumine antimoniate (treatment control group, 200 µg/mL, intralesional). No vehicle control group was included in the present study because castor oil additive is a nonionic surfactant commonly used as an excipient in pharmaceutical formulations, particularly in solubilizing poorly water-soluble drugs. It is widely used as an ingredient in cosmetics for its conditioning properties, based on occlusive action, reducing water loss, and having a nourishing effect on the skin, hair, and nails (Marwat et al. 2017; Khairani et al. 2022). In addition, although castor oil may be useful in the management of certain skin conditions such as urticaria, castor oil has no antileishmanial activity in vitro, with EC_50_ values of 208.07 ± 28.44 mg/mL. Likewise, castor oil applied on LC lesions in hamsters has not shown resolution of the lesions (data not shown).

Hamsters were treated daily with 40 µL of the corresponding formulation for 2 weeks, applying it directly over the lesion, while hamsters treated with meglumine antimoniate received treatment three times per week for 4 weeks. Lesion area was measured every week for 90 days with an electronic digital caliper. The body weight gain or loss was supervised every 2 weeks during the study. At the end of the study, hamsters were euthanized with an i.p. injection of ketamine (600 mg/kg) and xylazine (30 mg/kg). The experiments were carried out under normal laboratory lighting conditions. After necropsy, biopsies from healed and unhealed ulcers were removed and fixed in 10% buffered formalin, processed, and stained with hematoxylin–eosin (H-E) stain for histopathology analysis. The changes were analyzed under a standard optical microscope.

### Data analysis

The results are reported as mean values ± standard deviation. The LC_50_ and the EC_50_ were calculated by Probit analysis using GraphPad Prism 10 (San Diego, CA, USA). Both cytotoxicity and leishmanicidal activity were defined by ranges of values of LC_50_ and EC_50_ in terms of high, moderate, or low as follows: high cytotoxicity for LC_50_ values < 100 µM, moderate cytotoxicity for LC_50_ values > 100 µM but < 200 µM, and low cytotoxicity for LC_50_ values > 200 µM. For leishmanicidal activity, high activity corresponds to EC_50_ values < 50 µM, moderate activity corresponds to EC_50_ values > 50 µM but < 100 µM, and low activity corresponds to EC_50_ values > 100 µM. In addition, the cytotoxicity in U937 macrophages was correlated with the leishmanicidal activity by calculating the Selectivity Index (SI), using the equation: SI = LC_50_/EC_50_ (Indrayanto et al. 2021).

## Results

### In vitro cytotoxicity and antileishmanial activity of QUE, CUR, and PIP

The potential cytotoxic effects of compounds QUE, CUR, and PIP were assessed in uninfected U-937 cell macrophages using the MTT assay. None of the three compounds exhibited in vitro cytotoxicity for macrophages, as indicated by LC_50_ values exceeding 200 µM (Table 1).

**Table 1:** Cytotoxicity and antileishmanial activity of QUE, CUR, and PIP

Compound	LC_50_ (μM)^a^	EC_50_ (μM)^b^	SI^c^	Cytotoxicity	Leishmanicidal activity
Piperine	> 700.9	8.76 ± 1.40	> 80	Low	High
Curcumin	> 542.9	30.9 ± 0.3	> 17.4	Low	High
Quercetin	> 661.7	32 ± 2	> 20.6	Low	High
DOXO^d^	1.47 ± 0.06	NA^e^	NA^e^	High	NA^e^
AMB^f^	56.1 ± 5.8	0.32 ± 0.01	172.7	High	High
Castor oil	> 10%	> 10%	> 10%	Low	Low

Data represent the cytotoxic and effective concentration for each compound in µM. ^a^Median Lethal Concentration, ^b^Median Effective Concentration, ^c^Selectivity Index = LC_50_/EC_50_, ^d^Doxorubicin, ^e^No apply, ^f^Amphotericin B

Subsequently, the leishmanicidal efficacy of these compounds was investigated in U-937 macrophages infected with amastigotes of *L. braziliensis*. The results revealed a significant reduction in the intracellular viability of the evaluated *Leishmania* species following 48 h of treatment with all three compounds (Table 1). Notably, compound PIP demonstrated superior effectiveness in diminishing the intracellular viability of the parasite compared to compounds QUE and CUR. This is supported by the respective EC_50_ values: 8.76 ± 1.40 for PIP, 32 ± 2 for QUE, and 30.9 ± 0.3 for CUR (Table 1). As expected, AMB exhibited high activity against intracellular amastigotes of *L. braziliensis* but was also highly cytotoxic to uninfected U-937 cell macrophages.

The high SI highlighted the compounds’ favorable selectivity for *L. braziliensis* parasites over human macrophages, with compound PIP exhibiting particularly noteworthy selectivity compared to the other compounds.

### Therapeutic response of hamsters infected with *L. braziliensis* after treatment with topical formulations of CUR, PIP, and QUE

**Fig. 2:**
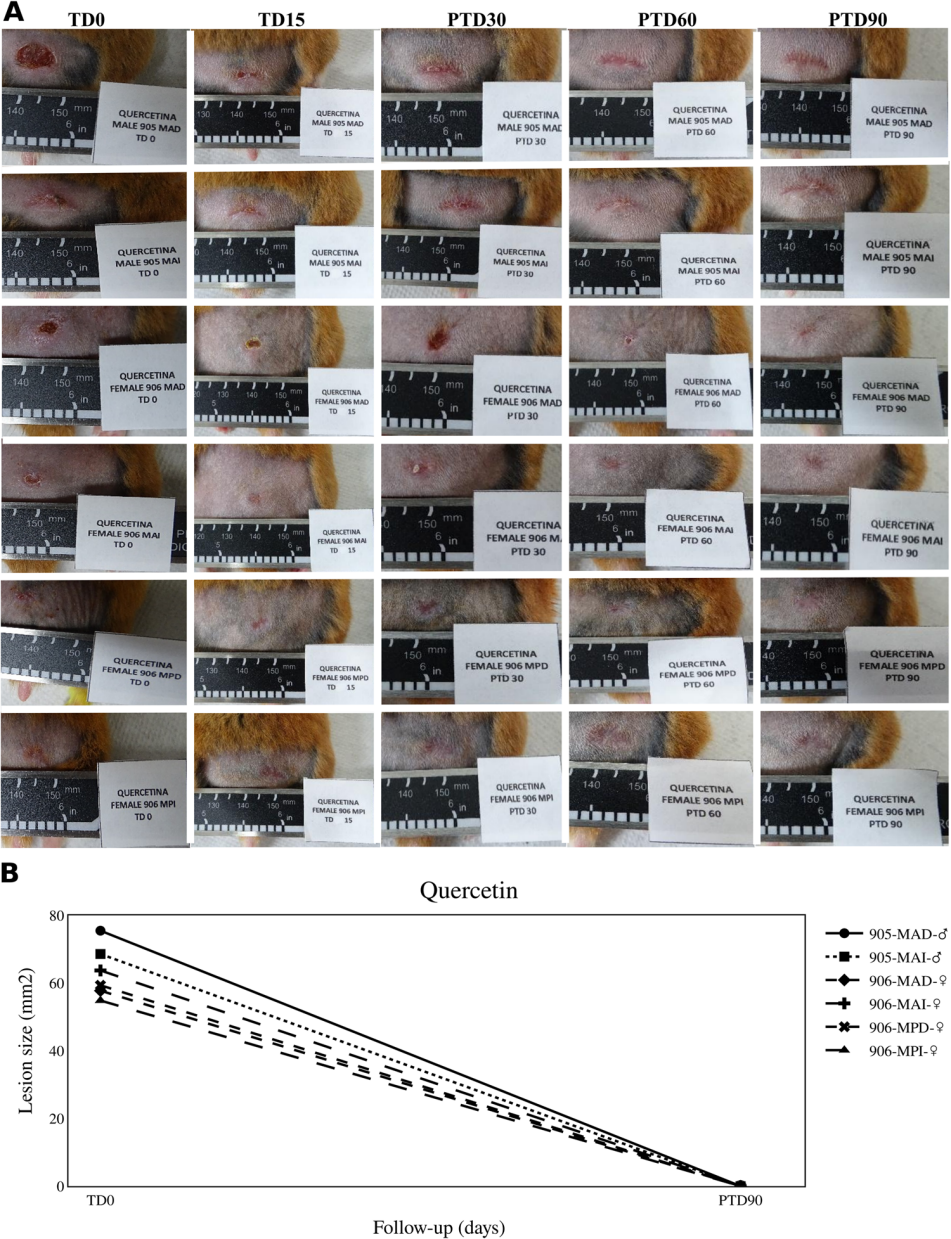
Therapeutic response to quercetin (QUE). **A** The photographs show the evolution of lesions in hamsters with CL and treated with QUE at 1 mg/day for 15 days. TD0, before treatment; TD15, last day of treatment; PTD30, 1 month after treatment ended; PTD60, 2 months after treatment; PTD90, 3 months after treatment. MAD, right forelimb; MAI, left forelimb; MPD, right hindlimb; and MPI, left hindlimb. **B** The graph shows the reduction of lesion size observed between the before treatment and the end of the study

All hamsters treated with QUE exhibited complete resolution of lesions, resulting in a 100% therapeutic response (Fig. 2).

**Fig. 3:**
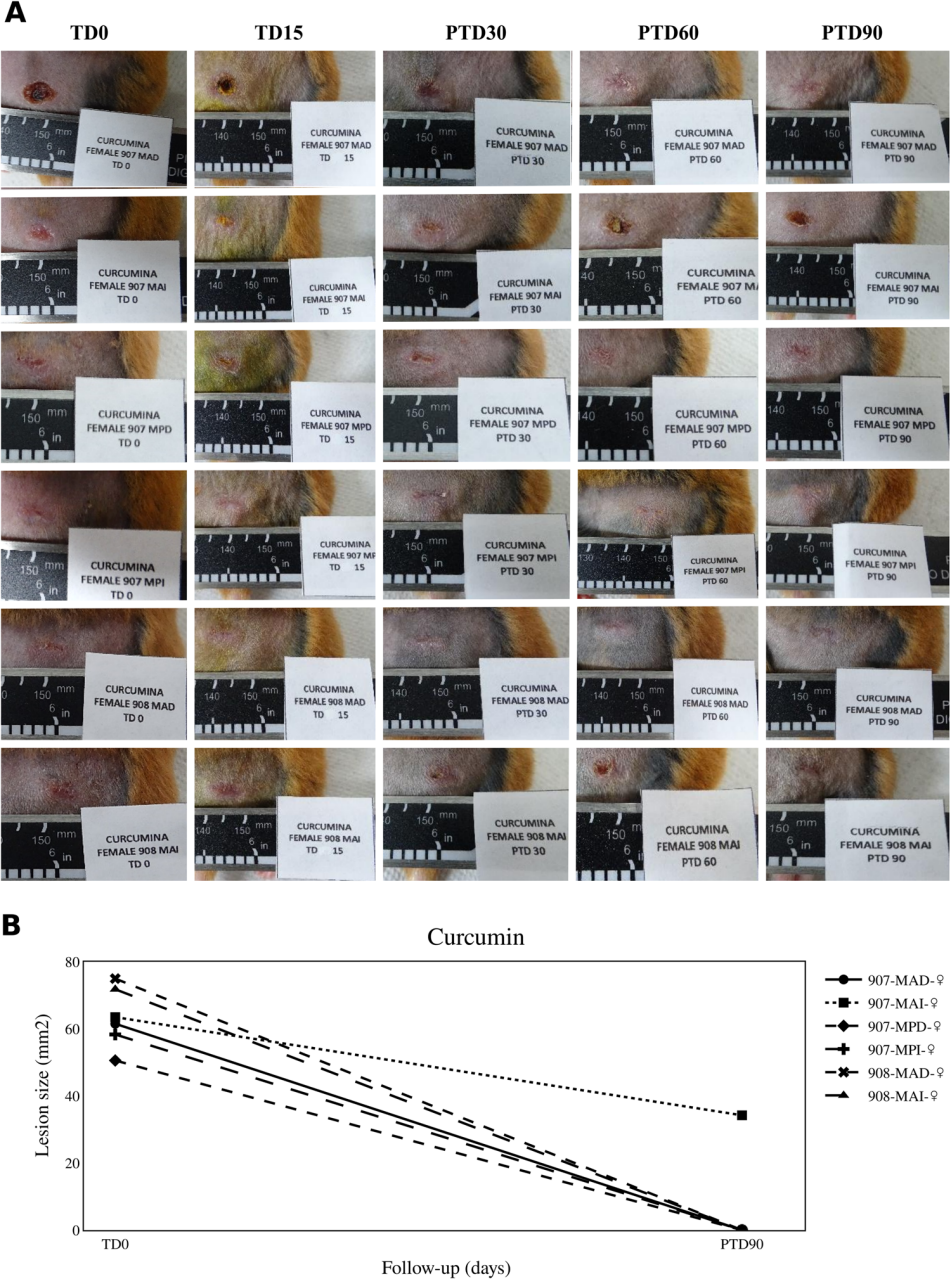
Therapeutic response to curcumin. **A** The photographs show the evolution of lesions in hamsters with CL and treated with curcumin (CUR) at 1.5 mg/day for 15 days. TD0, before treatment; TD15, last day of treatment; PTD30, 1 month after treatment ended; PTD60, 2 months after treatment; PTD90, 3 months after treatment. MAD, right forelimb; MAI, left forelimb; MPD, right hindlimb; and MPI, left hindlimb. **B** The graph shows the reduction of lesion size observed between the before treatment and the end of the study

In the group of hamsters treated with CUR, 5 hamsters were cured, and one hamster failed treatment (Fig. 3). So, the therapeutic response for CUR was 83%.

**Fig. 4:**
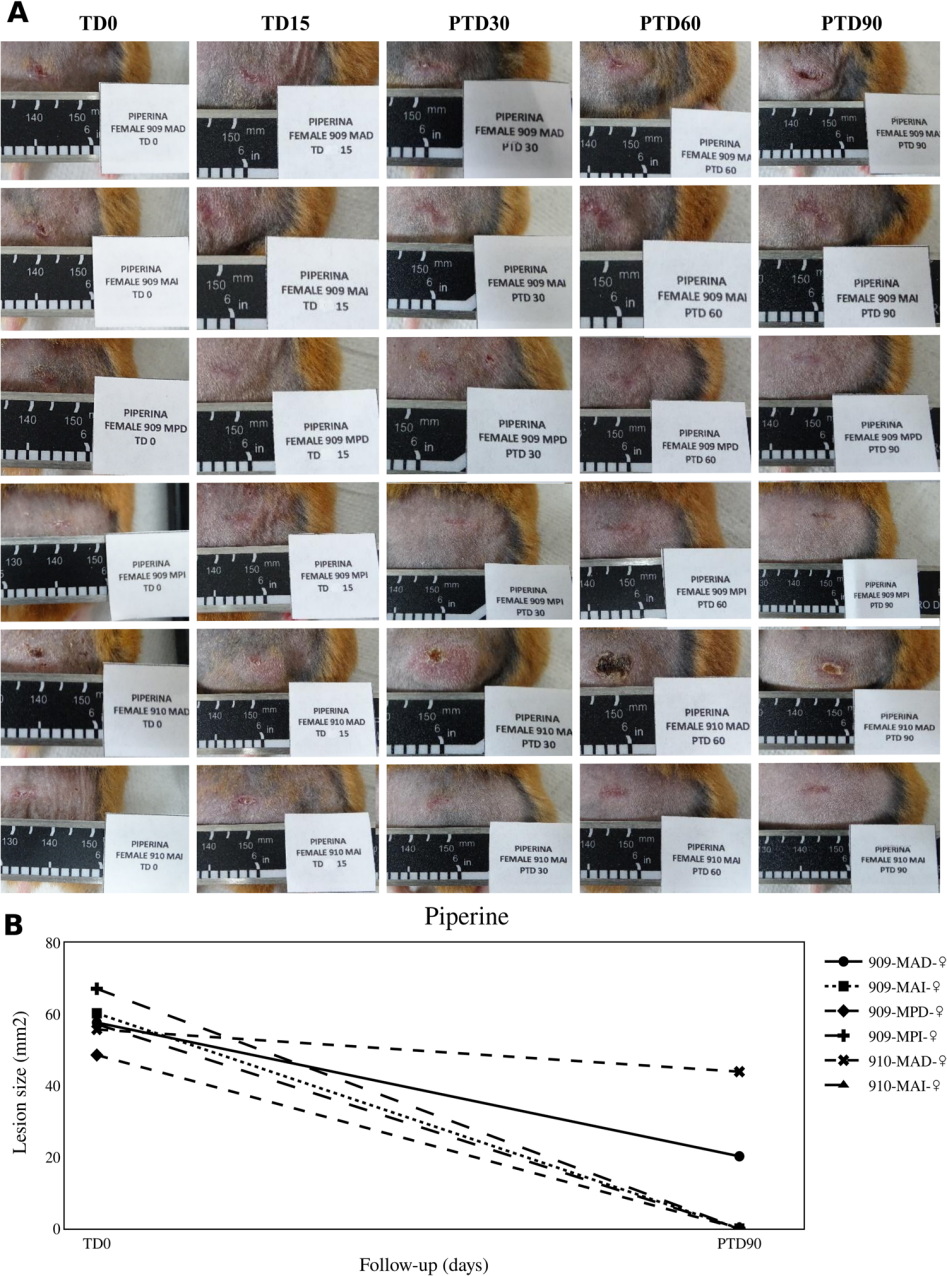
Therapeutic response to piperine. **A** The photographs show the evolution of lesions in hamsters with CL and treated with piperine at 2.5 mg/day for 15 days. TD0, before treatment; TD15, last day of treatment; PTD30, 1 month after treatment ended; PTD60, 2 months after treatment; PTD90, 3 months after treatment. MAD, right forelimb; MAI, left forelimb; MPD, right hindlimb; and MPI, left hindlimb. **B** The graph shows the reduction of lesion size observed between the before treatment and the end of the study

In turn, the therapeutic response to treatment with PIP was 67% (Fig. 4). Two hamsters showed a reduction in the lesion size of 65% and 21%. Although a higher dose was used with PIP, i.e., 2.5 mg/day than with QUE (1 mg/day) and CUR (1.5 mg/day), the therapeutic response was lower.

**Fig. 5:**
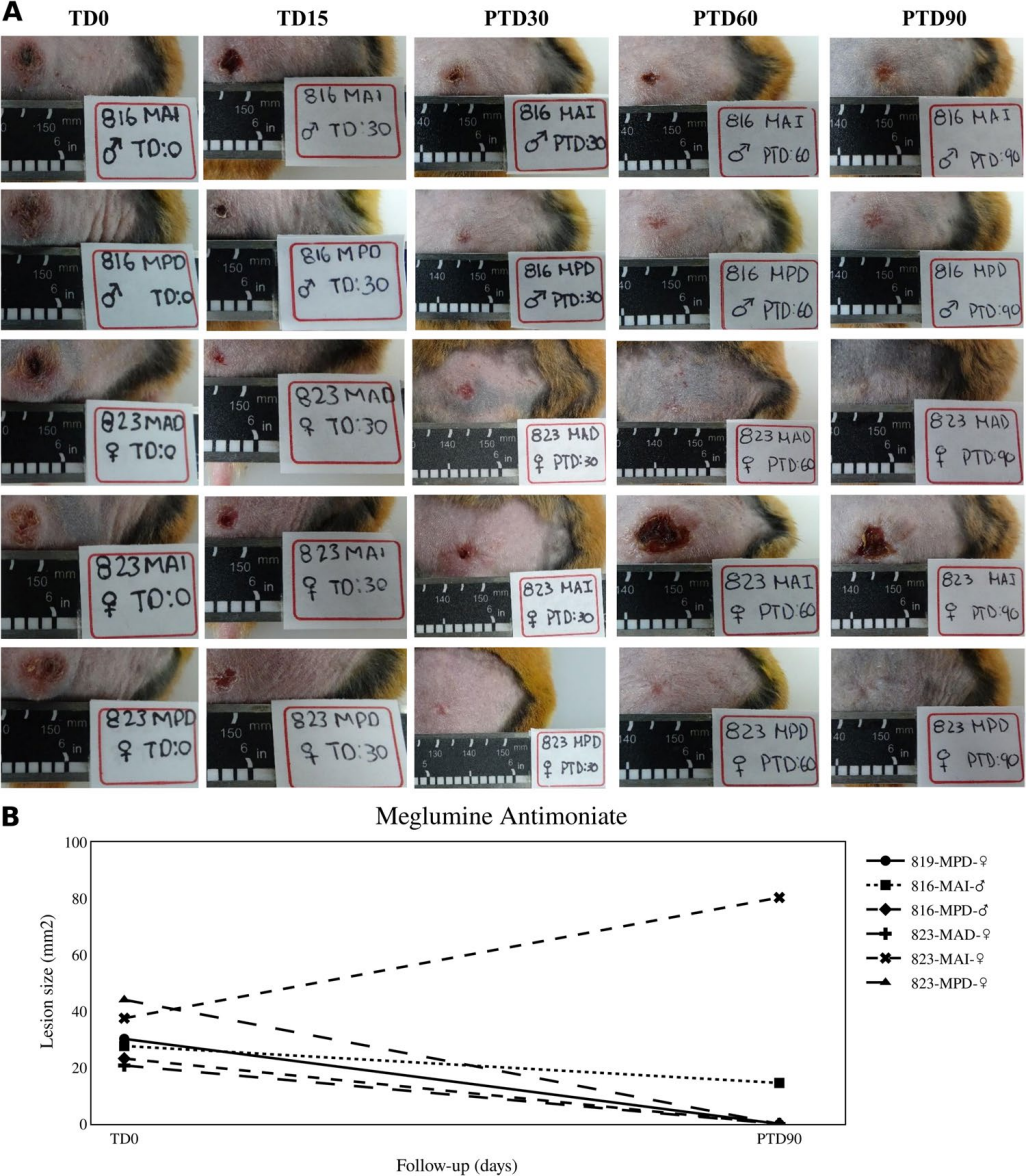
Therapeutic response to meglumine antimoniate. **A** The photographs show the evolution of lesions in hamsters with cutaneous leishmaniasis and treated with meglumine antimoniate at 200 µg/mL three times a week for 1 month. TD0, before treatment; TD15, last day of treatment; PTD30, 1 month after treatment ended; PTD60, 2 months after treatment; PTD90, 3 months after treatment. MAD, right forelimb; MAI, left forelimb; MPD, right hindlimb; and MPI, left hindlimb. **B** The graph shows the reduction of lesion size observed between the before treatment and the end of the study

Like the response shown by PIP, the therapeutic response to treatment with meglumine antimoniate was 67% with 4 hamsters cured after treatment (Fig. 5). Among the uncured hamsters, one of them showed a reduction in the lesion size of 14% while in the other hamster, its lesion increased in 80%.

### Histopathological analysis of healed ulcers from hamsters treated with QUE, PIP, and CUR emulsions

The histopathological findings of the healed skins for the three treatment groups were homogeneous and corresponded to a repair process following the reduction of the parasitic load. These findings consist of an epidermis with orthokeratotic hyperkeratosis and with espongiosis secondary to chronic inflammation in the process of resolution. In the dermis, the proliferation of connective tissue with abundant collagen deposition, numerous reactive fibroblasts, and fibroplasia along with neovascularization was evident. In the group treated with quercetin, the repair was at a more initial stage, which resulted in a higher degree of edema and inflammatory infiltration. Granulomas and amastigotes were not observed in the healed skins compared to the ulcerated skins (Table 2).

**Table 2:** Grades of lesion for the main histopathological findings of the unhealed and healed cut

Lesion	Active ulcer	Healed ulcer		
		PIP	CUR	QUE
Epidermis: orthokeratotic hyperkeratosis	0	2	2	2
Epidermis: spongiosis	0	2	1	2
Epidermis: ulcer	4	0	0	0
Dermis: neovascularization	1	2	4	2
Dermis: fibroplasia	0	2	2	3
Dermis: leukocytic infiltrate	4	2	2	3
Dermis: oedema	0	1	1	2
Dermis: granulomatous inflammation	5	0	0	0

The defined grades were as follows: 0, absent; 1, mild; 2, mild to moderate; 3, moderate; 4, moderate to severe; 5, severe aneous leishmaniasis ulcers

**Fig. 6:**
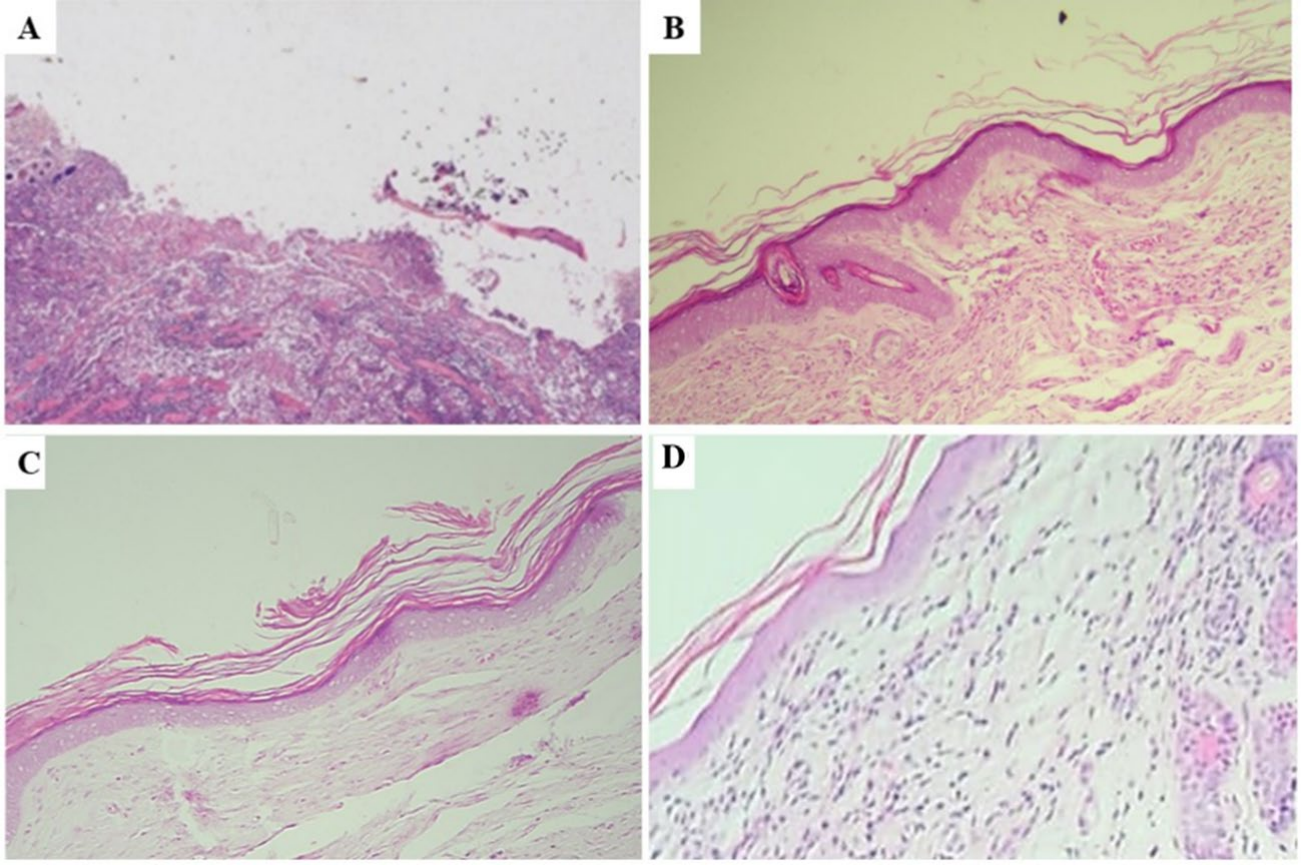
Histological features of cutaneous leishmaniasis ulcer before and after treatment with topical formulation of quercetin, piperine, and curcumin. **A** Extensive ulcer of a hamster experimentally inoculated with *L. braziliensis* on the skin of the dorsum; a deep diffuse mixed leukocytic infiltrate is evident in the dermis; H-E stain 100 × . **B** Healed hamster skin after topical treatment with QUE; in the dermis, there is evidence of increased deposition of collagen fibers characterized by swirls that are organized parallel to the epidermis, along with neovascularization and the presence of reactive fibroblasts. In addition, a mild diffuse leukocytic infiltrate with a lymphoplasmacytic predominance is seen; orthokeratotic hyperkeratosis and spongiosis are observed in the epidermis. No amastigotes are observed; H-E stain 100 × . **C** Healed hamster skin after topical treatment with PIP; same histopathological features of **B** in addition to lymphangiectasia; H-E stain 100 × . **D** Healed hamster skin after topical treatment with CUR; in the dermis, there is an observed increased neovascularization, edema, and the presence of reactive fibroblasts, scarring alopecia and loss of collagen fibers surrounding atrophic follicles hair; H-E stain 100 ×

The main histopathological finding in active ulcers (unhealed) was the presence of an intense inflammatory exudate with granulomas (Fig. 6A). In turn, healed CL ulcers treated with QUE, PIP, and CUR (Fig. 6B–D), respectively, showed re-epithelialization and neovascularization, a hallmark for the healing process, in addition to increased collagen deposition, fibroplasia, and inflammatory infiltrate.

**Fig. 7:**
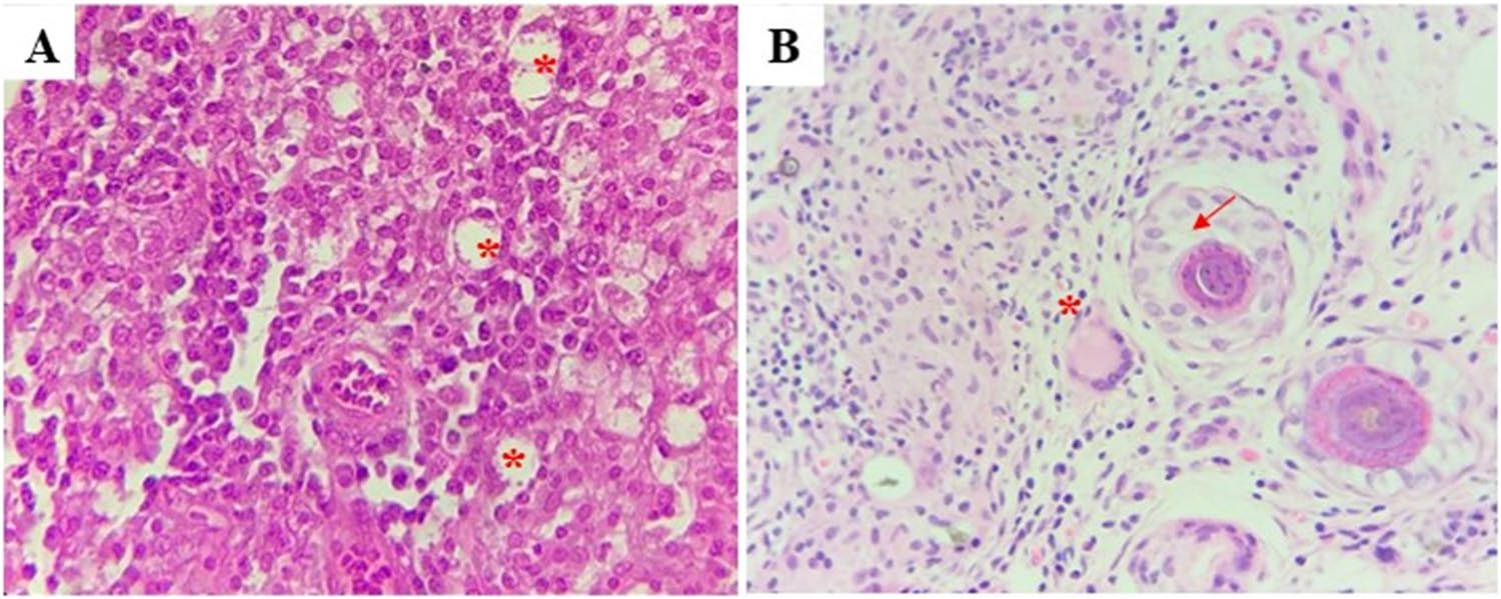
Histological features of unhealed and healed cutaneous leishmaniasis ulcer. **A** Dermis detail of the infiltrate, consisting mainly of activated macrophages and to a lesser extent epithelioid cells, lymphocytes, and plasmacytes; asterisk points the presence of amastigotes inside macrophages vacuoles; H-E stain 400 × . **B**. Detail of granuloma on deep dermis with apoptotic bodies, epithelioid cells, lymphocytes, plasmacytes, and multinucleated giant cell (asterisk); H-E

In active ulcers, inflammation observed in unhealed CL ulcers was mainly composed of epithelioid macrophages, neutrophils, lymphocytes, plasmacytes, and infected macrophages (Fig. 7A). On the other hand, in healed dermis, there is an observed extensive presence of apoptotic bodies and small vessels formed in the area closest to the epidermis (Fig. 7B).

### Toxicity

**Fig. 8:**
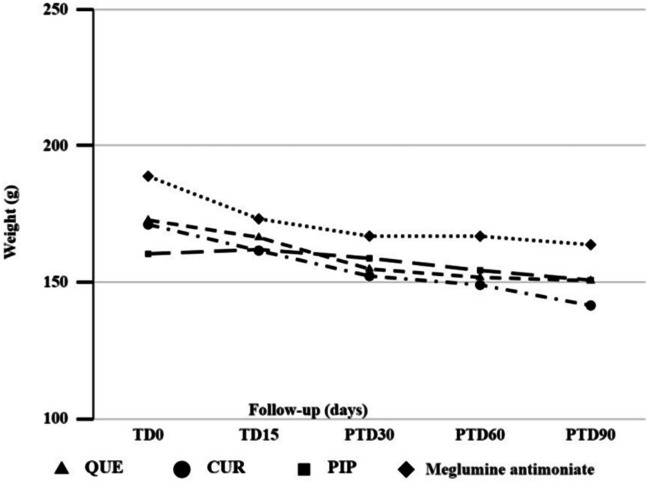
Body weight evolution of hamsters infected by *L. braziliensis* and treated with QUE, CUR, and PIP. The figure shows the weight in grams of hamsters during and after topical treatment with QUE, CUR, and PIP, vs meglumine antimoniate

There was a slight decrease in the body weight of hamsters after treatment (Fig. 8). Nonetheless, this loss did not compromise animal welfare.

## Discussion

Treatments against CL have been neglected, as most efforts have focused on visceral leishmaniasis (VL), the deadly form of the disease (Corman et al. 2023). However, the morbidity associated with CL is very high and the development of new treatments is required. It is important to note that, while CL often resolves spontaneously, there is a risk of progression to other forms of leishmaniasis, such as diffuse cutaneous leishmaniasis and mucocutaneous leishmaniasis. The variability in the duration and clinical course of CL underscores the need for careful monitoring and management of cases to prevent complications and ensure appropriate treatment (de Vries and Schallig 2022).

A general consensus is to use local treatment, including intralesional injection of sodium stibogluconate, heat treatment, and topical treatment or combinations of the aforementioned treatments. Among these, topical formulations could be a rational approach for the cure of patients with few, localized or non-complicated lesions caused by *Leishmania* spp. from the old and new world (Azim et al. 2021). In this study, we investigated the leishmanicidal activity of QUE, CUR, and PIP in both in vitro and in vivo models of *L. braziliensis* infection.

The study highlights the potential of these natural compounds as affordable and effective topical treatments for CL, addressing the need for alternative therapies.

Our results reveal a substantial decrease in the in vitro intracellular viability of *L. braziliensis* after 48 h of treatment with the three compounds. Assessing intracellular amastigotes is a clinically relevant model for evaluating antileishmanial drug efficacy, providing advantages such as insights into cellular well-being, cost-effectiveness, and eliminating the need for additional assays. Based on the criteria of (Indrayanto et al. 2021), the compounds evaluated exert high leishmanicidal activity. Furthermore, the obtained LC_50_ values on uninfected macrophages, exceeding 200 µM for all three compounds, indicate a non-cytotoxic profile, enhancing the promise of their therapeutic applications. Indrayanto et al. (2021) suggested that a model drug should have a relatively high LC_50_ and a very low EC_50_ to achieve a high SI of ≥ 10, making the compound worthy of further investigation (Indrayanto et al. 2021). The notable SI values observed, especially with compound PIP, underscore its potential as a selective and effective treatment option for *L. braziliensis* infections.

While the leishmanicidal effects of the investigated natural compounds are well-documented in the scientific literature, most publications have predominantly focused on the effects of QUE and CUR on the promastigote forms of others *Leishmania* species (Fonseca-Silva et al. 2013; Montrieux et al. 2014; Elamin et al. 2021; Albalawi et al. 2021b). In 2019, Cataneo et al. assessed the direct in vitro effect of the flavonoid QUE against *L. braziliensis*. They suggested that QUE’s antileishmanial effects on amastigotes involve activating Nrf2/HO-1, followed by modulating labile iron stores, resulting in depleted iron for the replication and survival of *L. braziliensis*. Recently, Santos et al. assessed the oral administration of QUE for the first time in hamsters infected with *L. braziliensis*, unveiling antiamastigote activity (EC_50_ of 21 ± 2.5 µM) (Dos Santos et al. 2022). A substantial decrease in macrophage viability was observed only at concentrations exceeding 640 µM, with an estimated EC_50_ of 478 ± 89 µM and a SI of 22, mirroring our study’s results. However, our research is differentiated because the focus is on exploring QUE as an option topical agent for CL caused by *L. braziliensis*. When applied directly to the skin, one can leverage its anti-inflammatory and antioxidant properties, as well as its effects on wound healing.

Regarding CUR, systematic reviews of this compound and its derivatives detail that the most common species of the *Leishmania* parasite used to evaluate their antileishmanial properties are *L. donovani*, *L. major*, and *L. amazonensis*, respectively. These reviews highlight that CUR and its derivatives could be considered an alternative and complementary source of valuable antileishmanial components against leishmaniasis, without causing significant toxicity (Saberi et al. 2021; Almadani et al. 2021). Notably, most investigations were also conducted at the promastigote stage rather than the more relevant intracellular amastigote stage (Saberi et al. 2021). Our work gains significance by specifically addressing the antileishmanial effects of CUR on *L. braziliensis*, providing valuable insights into its efficacy against this particular species. Based on our results, it is evident that CUR effectively inhibits the growth of the intracellular amastigote form of *L. braziliensis*, displaying moderate activity against the parasite (EC_50_ of 30.9 ± 0.3 µM), as per the classification. The SI value exceeds 10, categorizing it as a bioactive compound with promising potential. This finding contributes uniquely to the existing research landscape. In the study conducted by Pereira et al. (2021), the accumulation of CUR in amastigotes internalized by macrophages infected with *L. braziliensis* was observed. This finding is significant as the amastigotes themselves, internalized by the macrophages, can also be a direct target of the reactive oxygen species produced (Pinto et al. 2016; Pereira et al. 2021; Maciel et al. 2021).

The few studies of CUR with *L. braziliensis* only evaluated the in vitro effect of Photodynamic Therapy (PDT) using CUR as a photosensitizer (Pinto et al. 2016; Pereira et al. 2021; Maciel et al. 2021). The notable aspect according to the studies is the minimal recovery of parasites after PDT treatment, indicating the effectiveness of CUR-associated PDT in eliminating parasites and reducing parasite load with a single or serial PDT application. However, the authors note that under the evaluated conditions, when CUR and light were administered separately, the results were not satisfactory (Pinto et al. 2016; Pereira et al. 2021; Maciel et al. 2021)).

The leishmanicidal activity of PIP is the least documented among the three compounds under study. Until 2010, data were solely available for L. *donovani* (Kapil 1993; Raay et al. 1999; Veerareddy et al. 2004; Singh et al. 2010). In 2011, a study demonstrated the leishmanicidal effects of PIP and its derivatives on L. *amazonensis*, the causative agent of CL in the Americas, akin to *L. braziliensis* (Ferreira et al. 2011). This research underscored PIP’s activity against both promastigotes and amastigotes within infected macrophages, inducing mitochondrial alterations. Importantly, PIP exhibited non-toxicity towards macrophages, with an EC_50_ of 28 µM for the amastigote stage. In our study, PIP exhibited better antileishmanicidal activity and selectivity against *L. braziliensis* compared to the other evaluated compounds, with a EC_50_ value of 8.76 ± 1.4 µM and a corresponding SI value exceeding 80. These results represent a novel contribution, and to the best of our knowledge, this research marks the initial investigation into the effects of PIP in both in vitro and in vivo models against the *L. braziliensis* species associated with CL. PIP has also been employed as a bioenhancers to enhance the leishmanicidal efficacy of other compounds, including commonly used leishmanicidal drugs, yielding promising results (Vieira-Araújo et al. 2018; Sharifi et al. 2023). This opens the door for future considerations to assess the combination of the evaluated compounds as a strategy that surpasses the use of individual compounds.

If we consider the in vitro antileishmanial activity of commonly used drugs, such as meglumine antimoniate, amphotericin B, and miltefosine, against the amastigote stage of *L. braziliensis*, as reported in various studies (Morais-Teixeira et al. 2008; Zauli-Nascimento et al. 2010; Espada et al. 2017; Robledo et al. 2022), our findings reveal promising potential. The evaluated compounds, including CUR (11.4 ± 0.1), PIP (2.5 ± 0.4), and QUE (9.7 ± 0.6) µg/ml, demonstrate high antileishmanial activity, consistent with the mentioned drugs, but notably stand out for their low toxicity. While the correlation with clinical outcomes is not direct, and variations exist in the types of macrophages used for infection, our results clearly emphasize the potential of the compounds. Although, in general, amphotericin B proves to be more effective, its use is limited due to its high toxicity.

In our in vivo experimental hamster model of *L. braziliensis*-induced CL, a 15-day topical treatment with QUE formulated in castor oil achieved a remarkable 100% cure rate after 90 days. Similarly, CUR and PIP, also formulated in castor oil, exhibited success rates of 83% and 67%, respectively. Although the wound healing potential in our in vivo model does not linearly correlate with our in *vitro* results, these findings undoubtedly highlight the substantial potential of the three compounds evaluated for the topical treatment of CL caused by *L. braziliensis*. Improved therapeutic performance would have been anticipated with PIP, given its better leishmanicidal effects in vitro. However, QUE demonstrated superior efficacy in terms of therapeutic response. Even with a lower dose of QUE (1 mg/day) compared to PIP (2.5 mg/day) and CUR (1.5 mg/day), the therapeutic response was higher. This greater effectiveness of QUE in the evolution of lesions may be related to numerous factors, including the intrinsic pharmacological properties of each compound.

The healing of cutaneous wounds is a highly intricate process involving various molecular and cellular pathways (Almadani et al. 2021; Krizanova et al. 2022). Sakthianandeswaren et al. established a close association between resistance to CL and the host’s ability to heal a skin wound. The repair of the wound is the outcome that makes it possible to establish the cure of the CL. In other words, the response to treatment is essentially based on the clinical cure and not on the parasitological cure. Clinical cure typically refers to the resolution of skin lesions and symptoms. In contrast, the parasitological cure involves the absence of parasites in the samples taken from the site of the lesion, which means that a sample (scraping or biopsy) would have to be taken from the already healed tissue, which would damage the newly formed skin, in other words, to damage the skin again. Since the goal of the treatment is to achieve healing (scarring of the damaged skin), it does not make sense, nor is it ethical, to re-injure the healed skin by taking a sample of healed skin either by scraping or biopsy to ensure that there are no parasites in the scar. On the other hand, the occurrence of a sterile cure has been questioned for several years because it has been demonstrated that non-viable parasites or their genetic material can persist in scar tissue for years and decades without causing the reactivation of ulcers or other types of lesions (Mendonça et al. 2004). Other studies suggest that Leishmania major induces a sterile cure within the scars (Sghaier et al. 2022). Thus, the manifestation of cutaneous leishmaniasis does not depend on the parasite load but on the immune response triggered by the parasites (whether many or few) and the parasites'’ virulence to evade the immune response. If live parasites capable of activating the immune response persist, tissue repair cannot occur. All this evidence suggests that determining the parasite load is not a necessary factor in determining the response to treatment.

Our results show that QUE-treated skin exhibited healed lesions characterized by increased deposition of mature collagen fibers, organized swirls, neovascularization, and the presence of reactive fibroblasts. Notably, the absence of amastigotes in scar tissue and leukocytic infiltrate indicated the successful elimination of the parasite. These in vivo findings reaffirm the therapeutic potential of QUE for CL and corroborate previous studies in different CL and VL models. In Santos et al.’s study, it was shown that administering oral QUE (20 mg/kg; five times a week) to hamsters infected with *L. braziliensis*, starting 7 days after infection for 8 weeks, effectively controlled the lesion size and reduced the parasite load in both the lesion and the draining lymph node (Dos Santos et al. 2022). In another study, histopathological analysis revealed a reduction in inflammatory cell count, an increase in fibroblasts, and enhanced collagen deposition in tissue sections from mice infected with *L. major* and subjected to oral QUE treatment at a dosage of 50 mg/kg for 28 consecutive days (Almadani et al. 2021).

We observed similar findings in lesions treated with CUR and PIP in our in vivo model. As mentioned earlier, variations in the therapeutic response of these compounds could be linked to a combination of their pharmacological properties, differences in hamster susceptibility, or factors associated with the formulation.

While existing studies indicate the potential benefits of these compounds in wound healing (Barchitta et al. 2019; Kumari et al. 2022; Alsareii et al. 2023), there is a notable gap in in vivo research specifically exploring their therapeutic activity in the context of leishmaniasis. Our study investigates the therapeutic potential of CUR, QUE, and PIP in experimental leishmaniasis, focusing on *L. braziliensis*, offering valuable insights into the efficacy of these compounds as potential treatments. These findings are particularly promising for South American countries, where *L. braziliensis* is the primary species responsible for cases. As for the safety of the compounds, the animals experienced only a minor reduction in body weight following treatment with any of the compounds, and in no instance did it adversely affect the overall well-being of the animals.

## Conclusion

Our study demonstrates the promising therapeutic potential of the natural compounds QUE, CUR, and PIP as effective and affordable topical treatments for CL caused by *L. braziliensis*. These compounds exhibited significant leishmanicidal activity in both in vitro and in vivo models, with QUE particularly standing out for its superior efficacy in wound healing and lesion resolution. The successful outcomes in our experimental hamster model underscore the viability of QUE, CUR, and PIP as alternative and accessible treatments for CL, addressing the urgent need for effective options against this neglected form of leishmaniasis. Further research and clinical trials are warranted to validate and optimize their therapeutic applications in human cases of CL.

## Data Availability

No datasets were generated or analysed during the current study.

## References

[CR1] Aggarwal BB, Harikumar KB (2009). Potential therapeutic effects of curcumin, the anti-inflammatory agent, against neurodegenerative, cardiovascular, pulmonary, metabolic, autoimmune and neoplastic diseases. Int J Biochem Cell Biol.

[CR2] Albalawi AE, Alanazi AD, Sharifi I, Ezzatkhah F (2021). A systematic review of curcumin and its derivatives as valuable sources of antileishmanial agents. Acta Parasitol.

[CR3] Albalawi AE, Khalaf AK, Alyousif MS (2021). Fe3O4@piroctone olamine magnetic nanoparticles: synthesize and therapeutic potential in cutaneous leishmaniasis. Biomed Pharmacother.

[CR4] Almadani YH, Vorstenbosch J, Davison PG, Murphy AM (2021). Wound healing: a comprehensive review. Semin Plast Surg.

[CR5] Alsareii SA, Ahmad J, Umar A (2023). Enhanced in vivo wound healing efficacy of a novel piperine-containing bioactive hydrogel in excision wound rat model. Mol.

[CR6] Azeem M, Hanif M, Mahmood K (2023). An insight into anticancer, antioxidant, antimicrobial, antidiabetic and anti-inflammatory effects of quercetin: a review. Polym Bull.

[CR7] Azim M, Khan SA, Ullah S (2021). Therapeutic advances in the topical treatment of cutaneous leishmaniasis: a review. PLoS Negl Trop Dis.

[CR8] Barchitta M, Maugeri A, Favara G (2019). Nutrition and wound healing: an overview focusing on the beneficial effects of curcumin. Int J Mol Sci.

[CR9] Basmaciyan L, Azas N, Casanova M (2018). Different apoptosis pathways in Leishmania parasites. Cell Death Discov.

[CR10] Boots AW, Haenen GRMM, Bast A (2008). Health effects of quercetin: from antioxidant to nutraceutical. Eur J Pharmacol.

[CR11] Chauhan IS, Rao GS, Shankar J (2018). Chemoprevention of Leishmaniasis: in-vitro antiparasitic activity of dibenzalacetone, a synthetic curcumin analog leads to apoptotic cell death in Leishmania donovani. Parasitol Int.

[CR12] Corman HN, McNamara CW, Bakowski MA (2023). Drug discovery for cutaneous leishmaniasis: a review of developments in the past 15 years. Microorg.

[CR13] Das R, Roy A, Dutta N, Majumder HK (2008). Reactive oxygen species and imbalance of calcium homeostasis contributes to curcumin induced programmed cell death in Leishmania donovani. Apoptosis.

[CR14] de Morais-Teixeira E, de Carvalho AS, da Costa JCS (2008). In vitro and in vivo activity of meglumine antimoniate produced at Farmanguinhos-Fiocruz, Brazil, against Leishmania (Leishmania) amazonensis, L (L.) chagasi and L (Viannia) braziliensis. Mem Inst Oswaldo Cruz.

[CR15] de Vries HJC, Schallig HD (2022). Cutaneous Leishmaniasis: a 2022 updated narrative review into diagnosis and management developments. Am J Clin Dermatol.

[CR16] Dos Santos RF, Da Silva T, de Brito ACS (2022). Therapeutic effect of oral quercetin in hamsters infected with Leishmania Viannia braziliensis. Front Cell Infect Microbiol.

[CR17] Elamin M, Al-Olayan E, Abdel-Gaber R, Yehia RS (2021). Anti-proliferative and apoptosis induction activities of curcumin on Leishmania major. Rev Argent Microbiol.

[CR18] Espada CR, Ribeiro-Dias F, Dorta ML (2017). Susceptibility to miltefosine in Brazilian clinical isolates of Leishmania (Viannia) braziliensis. Am J Trop Med Hyg.

[CR19] Ferreira C, Soares DC, Barreto-Junior CB (2011). Leishmanicidal effects of piperine, its derivatives, and analogues on Leishmania amazonensis. Phytochemistry.

[CR20] Fonseca-Silva F, Inacio JDF, Canto-Cavalheiro MM, Almeida-Amaral EE (2011). Reactive oxygen species production and mitochondrial dysfunction contribute to quercetin induced death in Leishmania amazonensis. PLoS ONE.

[CR21] Fonseca-Silva F, Inacio JDF, Canto-Cavalheiro MM, Almeida-Amaral EE (2013). Reactive oxygen species production by quercetin causes the death of Leishmania amazonensis intracellular amastigotes. J Nat Prod.

[CR22] Gervazoni LFO, Barcellos GB, Ferreira-Paes T, Almeida-Amaral EE (2020). Use of natural products in Leishmaniasis chemotherapy: an overview. Front Chem.

[CR23] Haq I-U, Imran M, Nadeem M (2021). Piperine: a review of its biological effects. Phytother Res.

[CR24] Hussain Z, Thu HE, Amjad MW (2017). Exploring recent developments to improve antioxidant, anti-inflammatory and antimicrobial efficacy of curcumin: a review of new trends and future perspectives. Mater Sci Eng C Mater Biol Appl.

[CR25] Indrayanto G, Putra GS, Suhud F (2021). Validation of in-vitro bioassay methods: application in herbal drug research. Profiles Drug Subst Excip Relat Methodol.

[CR26] Kapil A (1993). Piperine: a potent inhibitor of Leishmania donovani promastigotes in vitro. Planta Med.

[CR27] Khairani S, Fauziah N, Lina Wiraswati H (2022). Piperine enhances the antimalarial activity of curcumin in plasmodium berghei ANKA-infected mice: A novel approach for malaria prophylaxis. Evid Based Complement Alternat Med.

[CR28] Khan H, Ullah H, Aschner M (2019). Neuroprotective effects of quercetin in alzheimer’s disease. Biomolecules.

[CR29] Krizanova O, Penesova A, Sokol J (2022). Signaling pathways in cutaneous wound healing. Front Physiol.

[CR30] Kumari A, Raina N, Wahi A (2022). Wound-healing effects of curcumin and its nanoformulations: a comprehensive review. Pharm.

[CR31] Maciel LTR, Marcolino LMC, Maciel FBS (2021). Effect of serial photodynamic therapy with curcumin on Leishmania braziliensis and Leishmania amazonensis promastigotes. RSD.

[CR32] Marwat SK, Rehman F, Khan EA, et al (2017) Review - Ricinus cmmunis - Ethnomedicinal uses and pharmacological activities. Pak J Pharm Sci. 30:1815–1827 29084706

[CR33] Mendonça MG, de Brito MEF, Rodrigues EHG (2004). Persistence of leishmania parasites in scars after clinical cure of American cutaneous leishmaniasis: is there a sterile cure?. J Infect Dis.

[CR34] Montrieux E, Perera WH, García M (2014). In vitro and in vivo activity of major constituents from Pluchea carolinensis against Leishmania amazonensis. Parasitol Res.

[CR35] Moorthi C, Kathiresan K (2013). Curcumin–Piperine/Curcumin–Quercetin/Curcumin–Silibinin dual drug-loaded nanoparticulate combination therapy: a novel approach to target and treat multidrug-resistant cancers. J Med Hypotheses Ideas.

[CR36] OPS (2013) Leishmaniasis en las Américas: Recomendaciones para el tratamiento. In: OPS. https://iris.paho.org/handle/10665.2/7704. Accessed 23 Nov 2023

[CR37] Papakyriakopoulou P, Velidakis N, Khattab E (2022). Potential pharmaceutical applications of quercetin in cardiovascular diseases. Pharm.

[CR38] Percie du Sert N, Ahluwalia A, Alam S (2020). Reporting animal research: explanation and elaboration for the ARRIVE guidelines 2.0. PLoS Biol.

[CR39] Percie du Sert N, Hurst V, Ahluwalia A (2020). The ARRIVE guidelines 2.0: updated guidelines for reporting animal research. PLoS Biol.

[CR40] Pereira AHC, Marcolino LMC, Pinto JG, Ferreira-Strixino J (2021). Evaluation of the photodynamic therapy with curcumin on L. braziliensis and L. major amastigotes. Antibiotics (Basel).

[CR41] Pinto JG, Fontana LC, de Oliveira MA (2016). In vitro evaluation of photodynamic therapy using curcumin on Leishmania major and Leishmania braziliensis. Lasers Med Sci.

[CR42] Pulido SA, Muñoz DL, Restrepo AM (2012). Improvement of the green fluorescent protein reporter system in Leishmania spp. for the in vitro and in vivo screening of antileishmanial drugs. Acta Trop.

[CR43] Raay B, Medda S, Mukhopadhyay S, Basu MK (1999). Targeting of piperine intercalated in mannose-coated liposomes in experimental leishmaniasis. Indian J Biochem Biophys.

[CR44] Rani R, Kumar S, Dilbaghi N, Kumar R (2020). Nanotechnology enabled the enhancement of antitrypanosomal activity of piperine against Trypanosoma evansi. Exp Parasitol.

[CR45] Rasmussen HB, Christensen SB, Kvist LP, Karazmi A (2000). A simple and efficient separation of the curcumins, the antiprotozoal constituents of Curcuma longa. Planta Med.

[CR46] Ray L, Karthik R, Srivastava V (2021). Efficient antileishmanial activity of amphotericin B and piperine entrapped in enteric coated guar gum nanoparticles. Drug Deliv Transl Res.

[CR47] Robledo SM, Carrillo LM, Daza A (2012). Cutaneous leishmaniasis in the dorsal skin of hamsters: a useful model for the screening of antileishmanial drugs. J vis Exp.

[CR48] Robledo SM, Murillo J, Arbeláez N (2022). Therapeutic efficacy of arnica in hamsters with cutaneous leishmaniasis caused by Leishmania braziliensis and L. tropica. Pharma.

[CR49] Roshanravan N, Askari SF, Fazelian S (2023). The roles of quercetin in diabetes mellitus and related metabolic disorders; special focus on the modulation of gut microbiota: a comprehensive review. Crit Rev Food Sci Nutr.

[CR50] Saberi R, Fakhar M, Asfaram S (2021). A systematic literature review of curcumin with promising antileishmanial activity. Infect Disord Drug Targets.

[CR51] Saini N, Chopra B, Dhingra AK (2023). Synergistic effect of piperine and its derivatives: a comprehensive review. Curr Drug Res Rev.

[CR52] Sarkar S, Mandal S, Sinha J (2002). Quercetin: critical evaluation as an antileishmanial agent in vivo in hamsters using different vesicular delivery modes. J Drug Target.

[CR53] Sghaier RM, Benhnini F, Guerfali FZ (2022). Healed lesions of human cutaneous leishmaniasis caused by Leishmania major do not shelter persistent residual parasites. Front Cell Infect Microbiol.

[CR54] Sharifi F, Mohamadi N, Afgar A, Oliaee RT (2023). Anti-leishmanial, immunomodulatory and additive potential effect of Piperine on Leishmania major: the in silico and in vitro study of Piperine and its combination. Exp Parasitol.

[CR55] Singh IP, Jain SK, Kaur A (2010). Synthesis and antileishmanial activity of piperoyl-amino acid conjugates. Eur J Med Chem.

[CR56] Sunila ES, Kuttan G (2004). Immunomodulatory and antitumor activity of Piper longum Linn. and piperine. J Ethnopharmacol.

[CR57] Thimmulappa RK, Mudnakudu-Nagaraju KK, Shivamallu C (2021). Antiviral and immunomodulatory activity of curcumin: a case for prophylactic therapy for COVID-19. Heliyon.

[CR58] Uddin SJ, Hasan MF, Afroz M (2021). Curcumin and its multi-target function against pain and inflammation: an update of pre-clinical data. Curr Drug Targets.

[CR59] Veerareddy PR, Vobalaboina V, Nahid A (2004). Formulation and evaluation of oil-in-water emulsions of piperine in visceral leishmaniasis. Pharmazie.

[CR60] Vieira-Araújo FM, MacedoRondon FC, Pinto Vieira ÍG (2018). Sinergism between alkaloids piperine and capsaicin with meglumine antimoniate against Leishmania infantum. Exp Parasitol.

[CR61] WHO World Health Organization (2023) Leishmaniasis. In: WHO. https://www.who.int/news-room/fact-sheets/detail/leishmaniasis. Accessed 23 Nov 2023

[CR62] Zauli-Nascimento RC, Miguel DC, Yokoyama-Yasunaka JKU (2010). In vitro sensitivity of Leishmania (Viannia) braziliensis and Leishmania (Leishmania) amazonensis Brazilian isolates to meglumine antimoniate and amphotericin B. Trop Med Int Health.

[CR63] Zou H, Ye H, Kamaraj R (2021). A review on pharmacological activities and synergistic effect of quercetin with small molecule agents. Phytomedicine.

